# Histopathological and Proteomics Analysis of Shrimp *Litopenaeus vannamei* Infected with *Ecytonucleospora hepatopenaei*

**DOI:** 10.3390/microorganisms13020402

**Published:** 2025-02-12

**Authors:** Ping Ni, Yingyao Ma, Bingxin Shi, Mengqiang Wang

**Affiliations:** 1 MOE Key Laboratory of Marine Genetics and Breeding, Shandong Key Laboratory of Marine Seed Industry (Preparatory), Qingdao Institute of Maritime Silk Road (Qingdao Institute of Blue Seed Industry), Ocean University of China, Qingdao 266003, China; 2 Hainan Key Laboratory of Tropical Aquatic Germplasm, Sanya Oceanographic Institution, Ocean University of China, Sanya 572024, China; 3 Southern Marine Science and Engineering Guangdong Laboratory (Guangzhou), Guangzhou 511458, China

**Keywords:** *Ecytonucleospora hepatopenaei*, histopathology, proteomics, *Litopenaeus vannamei*, shrimp disease surveillance

## Abstract

*Ecytonucleospora hepatopenaei* (EHP) is a microsporidian pathogen that primarily infects the hepatopancreas of *Litopenaeus vannamei*. Previous studies on EHP detection primarily focused on histology, in situ hybridization (ISH), and PCR, mainly concentrating on hepatopancreatic infections, with limited research on extra-hepatopancreatic tissues. This study investigates the pathogenic mechanisms of EHP infection in *L. vannamei* through molecular quantification, histopathology, and proteomics analysis. RT-qPCR was employed to examine the infection differences across various tissues at the molecular level, revealing that the hepatopancreas, stomach, midgut, muscle, gills, and antennal glands are susceptible tissues. Pathological analysis, combining H&E staining, Masson’s trichrome staining, and immunohistochemistry, identified the EHP-targeted sites at the tissue level. Masson’s staining effectively highlighted fibrosis, unveiling the histopathological characteristics of chronic EHP infection, while immunohistochemistry enhanced the specificity of EHP localization. The pathological features of EHP infection were primarily characterized by inflammation, cell degeneration and necrosis, and the accumulation of microsporidia in the cytoplasm. Proteomics analysis was used to interpret the histopathological findings, revealing significant enrichment of pathways related to inflammation, immune regulation, metabolism, and apoptosis regulation. These findings provide new insights into the infection mechanisms and tissue tropism of EHP.

## 1. Introduction

Since 2002, the widespread farming of *Litopenaeus vannamei* in Asia has contributed to *Ecytonucleospora hepatopenaei* (EHP) emerging as a significant pathogen [1], primarily infecting shrimp species within the genus *Penaeus*. EHP, a microsporidian pathogen, was first discovered and identified in the hepatopancreas of black tiger shrimp *Penaeus monodon* in Thailand in 2004 [2]. As a parasitic microsporidian, EHP spores are ovoid, measuring approximately 1.7 × 1.0 µm under scanning electron microscopy [3]. Clinically, shrimp infected with EHP exhibit slow growth, often accompanied by soft shells, lethargy, reduced feed intake, and empty midguts [4]. EHP proliferates predominantly in the epithelial cells of the hepatopancreas, impairing digestive function and rendering shrimp more susceptible to secondary bacterial and viral infections [5]. The lack of specific clinical signs distinguishing EHP infections from other diseases poses challenges for its monitoring and control. To date, no effective treatment has been established for EHP infections [6]. Histological examinations of infected individuals reveal significant degeneration of hepatopancreatic tubules, with basophilic inclusions associated with EHP developmental stages observed in epithelial cells, and large spore aggregations present within the tubular lumen [3].

Current diagnostic methods for EHP include histological examination, in situ hybridization, and PCR. Through hematoxylin–eosin (H&E) staining, abundant microsporidia can be observed in the hepatopancreatic tubular epithelial cells of infected *L. vannamei* [7]. Real-time quantitative PCR (RT-qPCR) has been used to detect EHP infections of *L. vannamei* [8]. However, histological detection has limitations. For instance, studies have found that when EHP is detectable in the hepatopancreas and intestine of infected individuals, it is absent in other organs [9]. Similarly, in pathological analyses of muscle microsporidiosis in *Palaemon carinicauda*, microsporidia were found exclusively in muscle tissues, while no evidence of infection was observed in the gills, stomach, intestine, heart, or hepatopancreas under light microscopy [10]. These findings highlight the challenges of relying solely on histological methods to confirm EHP infection and elucidate its mechanisms of pathogenesis.

Proteomic studies revealed that EHP infection significantly impacts immune-related and growth-related proteins in the hepatopancreas of *L. vannamei* [11]. Research indicates that EHP infection can notably induce the expression of various immune-related proteins while affecting the regulation of growth-associated proteins. Specifically, proteomic analysis demonstrates that EHP infection results in the downregulation of immune-related proteins, such as C-type lectins, hemocyanin, and glutathione transferase, suggesting that EHP infection may impair the innate immune functions of shrimp [12]. So, the present study integrates proteomics with histopathological approaches, in order to offer a novel perspective on the mechanisms of EHP infection and the identification of disease-resistant proteins. And the objectives of this study include identifying the susceptible tissues of *L. vannamei* to EHP infection using RT-qPCR, analyzing tissue damage and the specific aggregation sites of microsporidia at varying infection levels through histopathological examinations based on molecular quantification results, and integrating proteomic and histopathological analyses to explore the pathogenic mechanisms of EHP, the host’s immune responses, and potential disease-resistant proteins.

## 2. Materials and Methods

### 2.1. Infection Experiments and Sampling

The *L. vannamei* (average weight of 20 g and average length of 15 cm) utilized in the experiment were sourced from Hainan Lanyin Aquatic Seed Industry Technology Co., Ltd., Wenchang, China. The experimental subjects included 55 live *L. vannamei* individuals infected with EHP. Prior to the experiment, the shrimp were acclimated in the laboratory under controlled conditions, at a temperature of 26 ± 1 °C, pH of 8.0 ± 0.2, and salinity of 20‰, with continuous air. Water was partially renewed daily, with one-third replaced each morning and afternoon. The pathogens tested included other common pathogens such as white spot syndrome virus (WSSV), *Vibrio parahaemolyticus*, and Decapod iridescent virus (DIV1), all of which were negative. After a one-week acclimation period, the shrimps were prepared for subsequent infection trials. To enhance infection, an infection experiment was conducted using EHP-contaminated bait, with shrimp being fed a fixed quantity of EHP-infected bait during the three days preceding the experiment. The hepatopancreatic enterocystis bait used for the infection experiment was supplied by Hebei Xinhai Aquatic Seed Industry Technology Co., Ltd., Cangzhou, China.

Since the samples were already infected with EHP, which is less lethal compared to certain acute viral diseases, sampling began from the first day of the infection to obtain *L. vannamei* with varying degrees of infection. During the sampling process, live, moribund, and deceased shrimps were collected. A total of 25 surviving shrimps, 17 dying ones, and 13 deceased ones were collected. Each shrimp was dissected using sterilized scissors and forceps. Thirteen tissues were extracted from each specimen, including the gills, walking legs, hepatopancreas, eye stalks, anterior, middle and posterior midgut, rectum, heart, pyloric stomach, cardiac stomach, antennal glands, muscles, and gonads. The samples were quickly frozen using liquid nitrogen and stored at −80 °C for subsequent pathogen analysis. Another set of 10 tissues, including the gills, hepatopancreas, midgut, rectum, heart, pyloric stomach, cardiac stomach, antennal glands, muscles, and gonads, was collected from each shrimp and fixed in 4% paraformaldehyde for histopathological examination. Additionally, a separate hepatopancreas sample from each shrimp was rapidly frozen in liquid nitrogen and preserved at −80 °C for proteomic analysis.

### 2.2. Real-Time Fluorescence Quantitative PCR Detection of EHP

The construction of a standard curve for EHP quantification was initially performed. The plasmid containing EHP-SSU was extracted using the Mini Plasmid Extraction Kit (DP103, Tiangen, China), in accordance with the protocol provided in the instruction manual. The copy number of the recombinant plasmid was calculated using the following formula:

$$\mathrm{Copy}\;\mathrm{number}\left(\mathrm{copies}/\mu L\right)=\frac{\left[M\left(\mathrm{ng}/\mu L\right)\times 6.02\times 10^{23}\left(\mathrm{copies}/\mathrm{mol}\right)\times 10^{-9}\right]}{\left[N\left(\mathrm{bp}\right)\times 660\left(g/\mathrm{mol}/\mathrm{bp}\right)\right]}$$

where M represents the concentration of the recombinant plasmid and N is the number of base pairs in the EHP plasmid. The concentration of the recombinant plasmid was determined by measuring UV absorbance at 260 nm, and this measurement was subsequently converted to the plasmid DNA copy number. A series of dilution standards, ranging from 1 × 10^8^ to 1 × 10^2^ copies/μL of recombinant plasmid, were prepared by calculating the plasmid’s concentration in copy numbers followed by gradient dilution. RT-qPCR was then conducted to amplify DNA, targeting the causative SSU gene, with the gradient-diluted plasmids serving as templates and ddH_2_O used as the negative control. The experiments were set up in three parallels, and a standard regression line was derived from the average cycle threshold (Cq) values across the three runs. The primer and probe sequences for RT-qPCR are as follows: 157F (5′-AGTAAACTATGCCGACAA-3′), 157R (5′-AATTAAGCAGCACAATCC-3′), and TM-probe (FAM-TCCTGGTAGTGTCCTTCCGT-TAMRA). The reaction mixture consisted of 12.5 μL of 2× Premix Ex Taq (RR390A, Takara, Beijing, China), 1 μL of 157F (10 μM), 1 μL of 157R (10 μM), 0.5 μL of TM-probe (10 μM), and 1 μL of DNA template, with a total volume of 25 μL. The reaction cycle procedure was 30 s at 95 °C, followed by 40 cycles of 5 s at 95 °C, and 30 s at 60 °C.

### 2.3. Tissue Section Preparation

Samples were fixed in 4% paraformaldehyde for 12–24 h, rinsed with 1× PBS buffer, and, if needed, briefly stored in 70% ethanol. Following standard dehydration, clearing, and wax infiltration procedures, tissues were embedded in paraffin wax blocks. After being held overnight at −20 °C, the blocks were sectioned into 5 μm slices using a rotary microtome, then spread, adhered, and dried. H&E staining was applied to the treated tissue sections. H&E staining was conducted with H&E Staining Kit (C0105S, Beyotime, Nantong, China), and the sections were then examined under the Leica DM2500 LED fluorescent orthotropic microscope (Leica DM2500 LED, Leica Microsystems, Wetzlar, Germany). Masson staining was applied to the treated tissue sections. Staining was performed using Masson Trichrome Staining Solution Kit (G1006, Servicebio, Wuhan, China), and observations were subsequently made with the Leica Aperio GT450 digital pathology system. For the immunohistochemical observation, a custom rabbit-derived polyclonal antibody against Spore Wall Protein 1 (SWP1) (WG-05378D, ABclonal, Wuhan, China) was used as the primary antibody, and HRP-conjugated goat anti-rabbit IgG (H + L) (AS014, ABclonal, Wuhan, China) was used as the secondary antibody. After antigen retrieval, blocking, antibody incubation, and DAB staining, the sections were analyzed using the Leica Aperio GT450 digital pathology system.

### 2.4. Proteomic Sample Preparation

Hepatopancreas samples with copy numbers quantified through a quantitative assay were organized into three groups, including a blank control group, a low-copy group, and a high-copy group, each comprising three biological replicates. Protein extraction was performed by suspending the samples in a lysis buffer containing 1% sodium deoxycholate (SDS), 8 M urea, and an appropriate 1× protease inhibitor cocktail to inhibit protease activity. After homogenization and centrifugation, the protein concentration in the supernatant was measured using the bicinchoninic acid (BCA) method with a BCA Protein Assay Kit, following the manufacturer’s protocol. The sample preparation process includes protein denaturation, reduction, alkylation, tryptic digestion, and peptide cleanup. A commercially available iST Sample Preparation Kit (PreOmics, Planegg, Germany) was utilized following the manufacturer’s protocols. Peptides obtained after preprocessing were reconstituted in Solution A (0.1% formic acid in water) and analyzed using LC-MS/MS. The analysis was performed on a timsTOF Pro2 mass spectrometer (Bruker Daltonics, Billerica, MA, USA) coupled to an UltiMate 3000 system (Thermo Fisher Scientific, Waltham, MA, USA). DIA data acquisition was carried out in diaPASEF mode. The DIA data were subsequently analyzed using Spectronaut 18 with default parameters (BGS Factory Settings). Subsequently, correlation analysis of the samples was performed using Principal Component Analysis (PCA) with the gmodels R package (http://www.r-project.org/, accessed in August 2024). PCA was calculated to assess the reproducibility among the samples and assist in identifying and excluding outliers. Differentially expressed proteins (DEPs) were analyzed with Student’s *t* Test and Benjamini and Hochberg (BH). After that, DEPs were filtered with the selection criteria of fold change >1.5 and *p* value < 0.05. Based on the DEPs, an enrichment analysis was performed. The DEPs were mapped to the Gene Ontology (GO) terms using the GO database, and the number of proteins associated with each term was calculated to generate a list of proteins and their corresponding counts for each GO function. Subsequently, hypergeometric tests were applied to identify GO terms that were significantly enriched in the DEPs compared to the entire background protein set. For pathway enrichment analysis, the KEGG pathway database was used, and hypergeometric tests were again applied to identify pathways that were significantly enriched in the DEPs compared to the background protein set.

## 3. Results

### 3.1. Quantification of EHP Load via RT-qPCR

**Figure 1:**
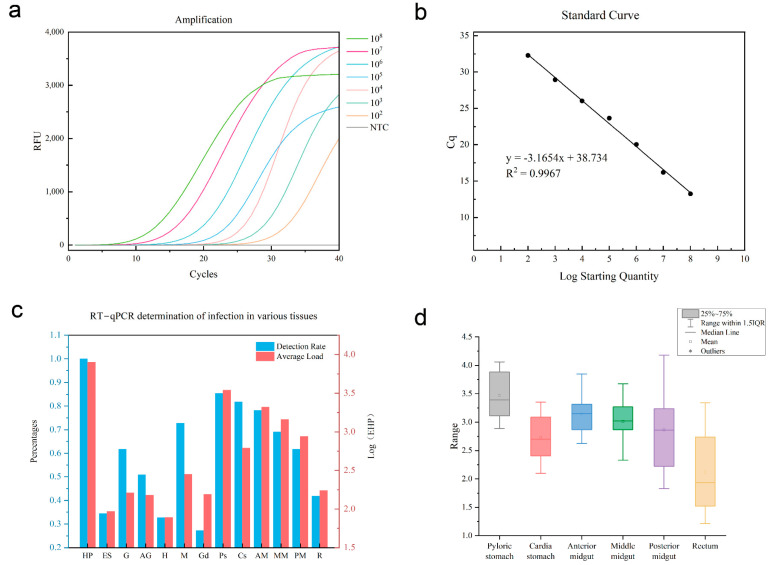
Construction of EHP standard curve and EHP loading of each tissue determined by RT-qPCR. (**a**) EHP recombinant plasmid amplification curve (10^8^–10^2^ copies/μL); (**b**) EHP recombinant plasmid standard curve; (**c**) RT-qPCR for EHP loading on different tissues and detection rate. HP: hepatopancreas. ES: eyestalk. G: gill. AG: antennal gland. H: heart. M: muscle. Gd: gonad. Ps: pyloricstomach. Cs: cardiastomach. AM: anterior midgut. MM: middle midgut. PM: Posterior midgut. R: rectum. (**d**) EHP loading in various tissues of the digestive tract.

RT-qPCR was conducted using a dilution series of standard EHP plasmid samples (ranging from 1 × 10^8^ to 1 × 10^2^ copies/μL of recombinant plasmid). The amplification curve is displayed in Figure 1a, with sequential mean Cq values of 13.24, 16.19, 20.02, 23.66, 26.02, 28.93, and 32.29. These Cq values are plotted as vertical coordinates, while the logarithm of the copy number concentration serves as the horizontal coordinate to create a standard regression line, as shown in Figure 1b. The regression equation for the standard curve is y = −3.1654x + 38.734, where x represents Log starting quantity and y represents Cq. Correlation coefficient R^2^ = 0.9967. These results indicate high confidence in the standard curve, demonstrating a good linear relationship between the logarithm of the plasmid standard’s copy number concentration and the Cq value.

RT-qPCR was conducted on DNA extracted from each tissue to determine the corresponding Cq values, and the relative copy number for each tissue was calculated using the standard curve equation. The load values are represented as the logarithmic values of EHP. The RT-qPCR assay results indicated that EHP in shrimp infected various tissues, including the eyestalks, heart, hepatopancreas, pyloric stomach, cardiac stomach, muscle, anterior, middle and posterior midgut, rectum, antennal glands, and gonads in shrimps. Notably, EHP was absent only in the walking leg. The EHP detection rates and loads across 13 different tissues in 55 sets of shrimp tissue samples are shown in Figure 1c. Additionally, Table 1 presents a comparison of EHP loads in the same tissue under three shrimp conditions, including surviving, dying, and dead. The detection rates of EHP in shrimp organs vary significantly with health status. In live shrimp, detection rates are low, especially in the eye stalk, heart, and antennal gland, and the detection rate was 28%, 36%, and 44%, respectively. As shrimp health declines, the detection rate of dying shrimp did not change much, and even the tissue detection rate decreased, especially in the heart and rectum. In dead shrimp, detection rates are generally higher, with some organs such as the pyloric stomach, cardia stomach, heart, and anterior midgut showing rates near or exceeding 90%. Except for eye stalk, heart, and gonad, the detection rate of other tissues was more than 50%. EHP loads in live shrimp are low, particularly in the eyestalk, heart, and gill, with values between 1.05 and 1.89. With the deterioration of the health status of the shrimp, the load of dying shrimp increased, and the load of some tissues such as gill and pyloric stomach reached more than 2.45. The load of dead shrimps was generally higher than live shrimps, especially in hepatopancreas, pyloric stomach, and midgut, which were between 3.32 and 4.52, indicating a significant increase in the load of all organs in the state of death.

Given that EHP infection in shrimp primarily impairs digestion and absorption functions, tissues from the digestive tract were specifically evaluated in the quantitative measurements. These included six types of tissues: the pyloric stomach, cardiac stomach, anterior midgut, middle midgut, posterior midgut, and rectum. After excluding shrimp with uninfected digestive tracts, 23 groups of infected shrimps were analyzed (Figure 1d). Among these six tissues, the EHP load was the highest in the pyloric stomach and the lowest in the rectum. The load in the cardiac stomach was significantly lower than that in the pyloric stomach and was generally lower than that in each part of the midgut. As for the load in the midgut, it gradually decreased from the anterior part to the posterior part.

### 3.2. Histopathology Under H&E Staining

**Figure 2:**
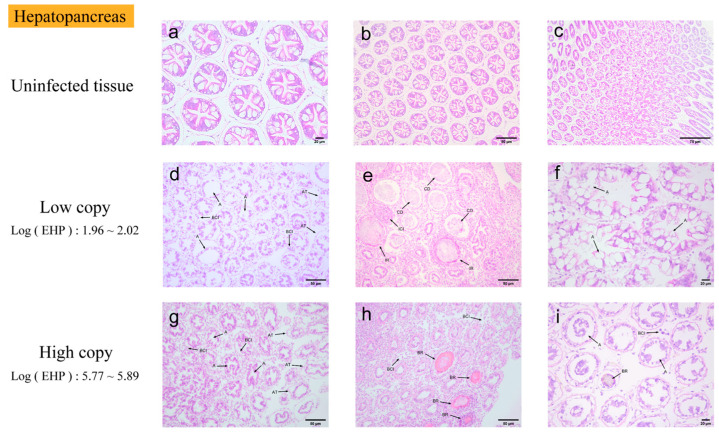
H&E staining of the hepatopancreas: (**a**–**c**) uninfected hepatopancreas; (**d**–**f**) low-load hepatopancreas; (**g**–**i**) high-load hepatopancreas. A: severe atrophy of hepatopancreas; vacuolation in B and R cells; extensive detachment of renal tubular cells. AT: abscission tissue. BCI: blood cell infiltration. CD: loss of tubular structure, absence of lipids and cell differentiation, lumen filled with cellular debris. IR: inflammatory response. ICI: focal accumulation of inflammatory cells. BR: blackening reaction.

Hepatopancreas. The histopathological characteristics of the hepatopancreas infected with EHP are illustrated in Figure 2. Microscopic examination of hepatopancreatic tissues exhibiting low EHP loads revealed significant atrophy of the hepatopancreas, vacuolization of B and R cells, and widespread detachment of renal tubular cells. Furthermore, loose tissue detachment and extensive hematopoietic infiltration were observed at multiple sites. Concurrently, there is a loss of tubular structure, absence of lipids, and a lack of differentiation among cell populations, with the lumen filled with necrotic debris. An inflammatory response commenced, characterized by surrounding hematopoietic infiltration, and numerous inflammatory cells were observed to infiltrate in focal areas of severe inflammation. The hepatopancreatic tissues with high EHP loads exhibited a more pronounced inflammatory response compared to those with low EHP loads, including a notable blackening reaction. However, no distinct microsporidia were detected.

**Figure 3:**
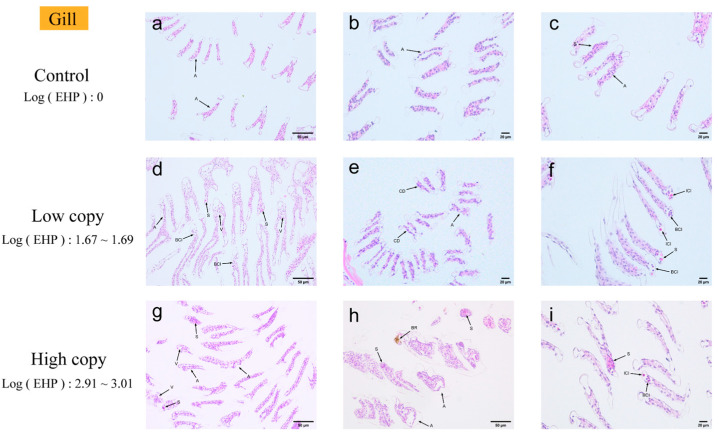
H&E staining of the gills; (**a**–**c**) negative gill; (**d**–**f**) low-load gill; (**g**–**i**) high-load gill. A: gill filament atrophy. V: vacuolation in the gill chamber. S: microsporidian presence. BCI: blood cell infiltration. CD: cellular degeneration. ICI: inflammatory cell infiltration. BR: blackening reaction.

Gill. Based on the quantitative experimental results, the gills were judged to be EHP-sensitive tissues (Figure 3). Microscopic examination of gill tissues with zero EHP load revealed signs of gill atrophy. In contrast, gill tissues with low EHP loads exhibited not only atrophy of the gill filaments but also the presence of vacuoles and infiltration of hemocytes within the gill lumen. EHP microsporidia were identifiable under high magnification. Additionally, the gill cells displayed signs of cellular degeneration along with multiple areas of inflammatory cell infiltration. In gill tissues affected by high EHP loads, there was a marked presence of numerous microsporidia, heightened inflammation, and increased infiltration of inflammatory cells, accompanied by a notable darkening reaction when compared to the low-copy group.

**Figure 4:**
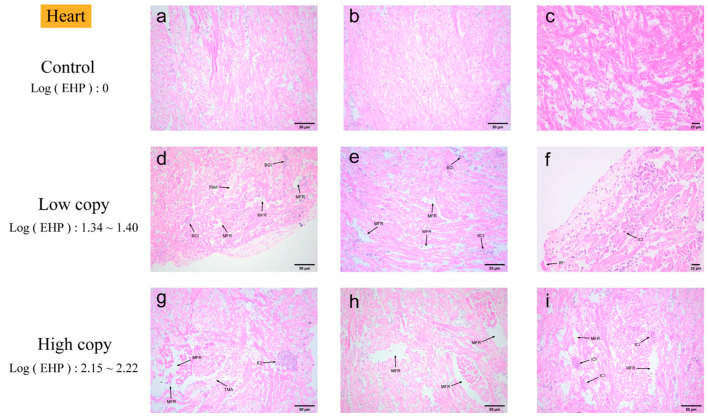
H&E staining of the hearts: (**a**–**c**) negative heart; (**d**–**f**) low-load heart; (**g**–**i**) high-load heart. MFR: muscle fiber rupture. TMA: transverse muscle ablation. BCI: blood cell infiltration. ICI: inflammatory cell infiltration. IR: inflammatory response.

Heart. Quantitative analysis indicated a low level of EHP infection in the heart, accompanied by some pathological response (Figure 4). No significant pathological changes were observed in heart tissue with zero EHP load. In heart tissue with low EHP load, microscopy revealed myocardial fiber rupture and rhabdomyolysis in lighter-stained areas, accompanied by blood and inflammatory cell infiltration. In heart tissue with high EHP load, an intensified inflammatory response was observed, with focal clusters of inflammatory cells in multiple areas.

**Figure 5:**
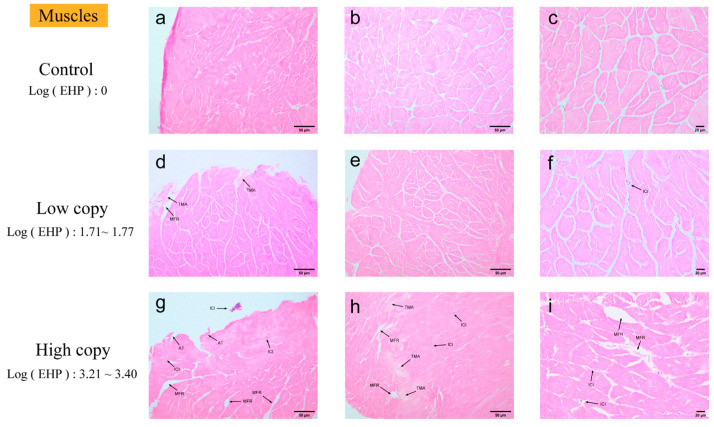
H&E staining of the muscles; (**a**–**c**) negative muscle; (**d**–**f**) low-load muscle; (**g**–**i**) high-load muscle. MFR: muscle fiber rupture. TMA: transverse muscle ablation. ICI: inflammatory cell infiltration with focal accumulation. AT: muscle fiber rupture with partial detachment of muscle tissue.

Muscle. According to the quantitative analysis, muscle tissue is identified as one of the tissues susceptible to EHP (Figure 5). No significant pathological changes were observed in muscle tissue with zero EHP load. In low EHP load muscle tissue, microscopy revealed focal muscle fiber rupture, rhabdomyolysis in lighter-stained areas, and initial inflammatory cell infiltration. These findings intensified with increased EHP load, but no microsporidian aggregation was observed.

**Figure 6:**
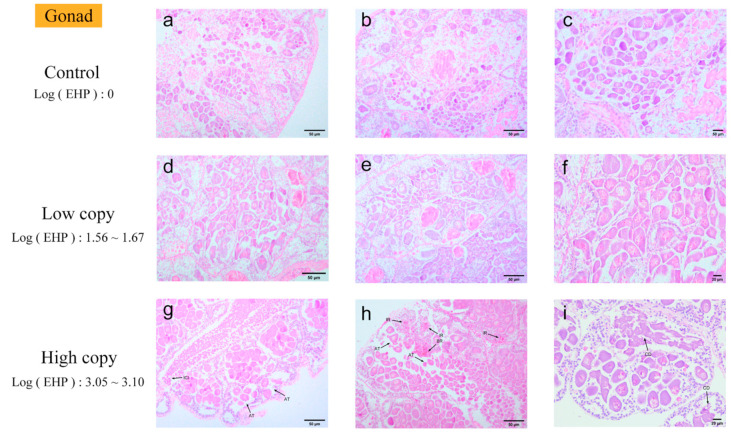
H&E staining of the gonads: (**a**–**c**) negative gonad; (**d**–**f**) low-load gonad; (**g**–**i**) high-load gonad. AT: loosely detached oocytes. ICI: inflammatory cell infiltration. BR: blackening reaction. IC: inflammatory response. CD: liquefactive necrosis of oocytes.

Gonad. Quantitative analyses indicate that EHP could infect the gonads. Tissue damage was observed in the gonads infected with EHP (Figure 6). In gonadal tissues with zero and low EHP loads, no significant pathological changes were observed under microscopic examination. In contrast, gonadal tissues with high EHP loads exhibited loose detachment of oocytes and the onset of an inflammatory response, characterized by inflammatory cell infiltration in multiple areas, with some regions showing signs of blackening. Additionally, liquefactive necrosis of oocytes was noted in the affected lesions.

**Figure 7:**
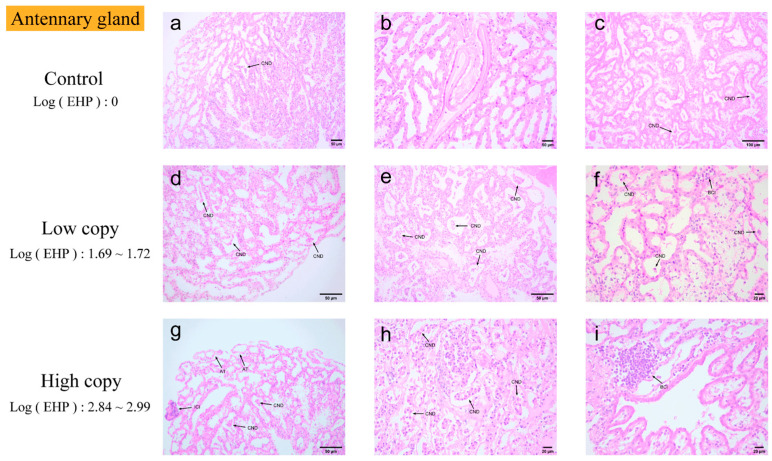
H&E staining of the antennal glands: (**a**–**c**) negative antennal gland; (**d**–**f**) low-load antennal gland; (**g**–**i**) high-load antennal gland. CND: minor necrosis and detachment of antennal gland cells, forming clusters. BCI: blood cell infiltration. ICI: extensive inflammatory cell infiltration with focal accumulation. AT: abscission tissue.

Antennal gland. In quantitative experiments, antennal glands were identified as tissues susceptible to EHP infection (Figure 7). In antennal gland tissue with zero EHP load, slight cellular necrosis and shedding were observed. In low EHP load antennal gland tissue, blood cell infiltration began to appear. With high EHP load, pathological changes became more pronounced. Microscopically, cells appeared deformed as EHP copy numbers increased, showing significant focal clusters of inflammatory cell infiltration and blood cell infiltration. Additionally, multiple areas exhibited tissue loosening and shedding.

**Figure 8:**
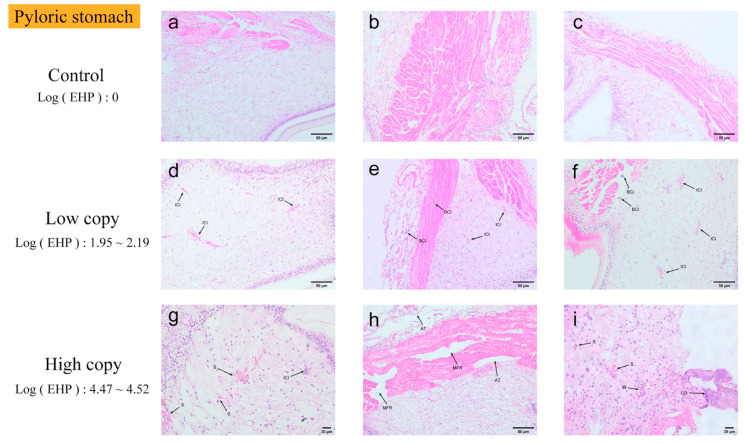
H&E staining of the pyloric stomachs: (**a**–**c**) negative pyloric stomach; (**d**–**f**) low-load pyloric stomach; (**g**–**i**) high-load pyloric stomach. ICI: inflammatory cell infiltration. BCI: blood cell infiltration. S: extensive microsporidian presence. MFR: muscle layer fiber rupture. TA: detachment between connective tissue layer and muscle layer. IR: inflammatory response. CD: extensive cell necrosis in connective tissue.

**Figure 9:**
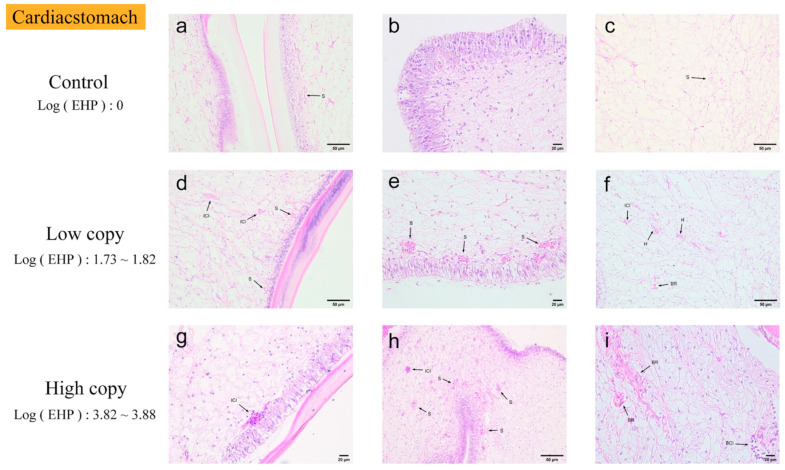
H&E staining of the cardiac stomachs: (**a**–**c**) negative cardiac stomach; (**d**–**f**) low-load cardiac stomach; (**g**–**i**) high-load cardiac stomach. S: microsporidian spores. ICI: inflammatory cell infiltration. H: holes in connective tissue. BR: blackening reaction. BCI: blood cell infiltration.

Pyloric and cardiac stomach. Based on the analysis of the quantitative experimental results, the stomach was found to be highly susceptible to infection (Figure 8 and Figure 9). In pyloric gastric tissues with low EHP loads, inflammatory cells and hematopoietic infiltration were observed. In contrast, pyloric gastric tissues with high EHP loads exhibited more severe tissue damage. In addition to the inflammatory response, a significant accumulation of microsporidia was seen as the infection level increased. Moreover, the muscle fibers of the muscular layer surrounding the pyloric stomach were broken, and the connective tissue layer was detached from the muscular layer in multiple areas. In the most severely affected regions, numerous cells in the connective tissue were necrotic. The pathological features of the cardiac stomach were comparable to those of the pyloric stomach. However, unlike the pyloric stomach, even at an EHP load of zero, trace amounts of microsporidia could already be observed in the cardiac stomach tissue. At low EHP loads, a melanotic reaction was observed, and the connective tissue appeared hollow. At higher loads, microscopic examination revealed an intensified inflammatory response due to the worsening infection, with microsporidian aggregates observed in several regions.

**Figure 10:**
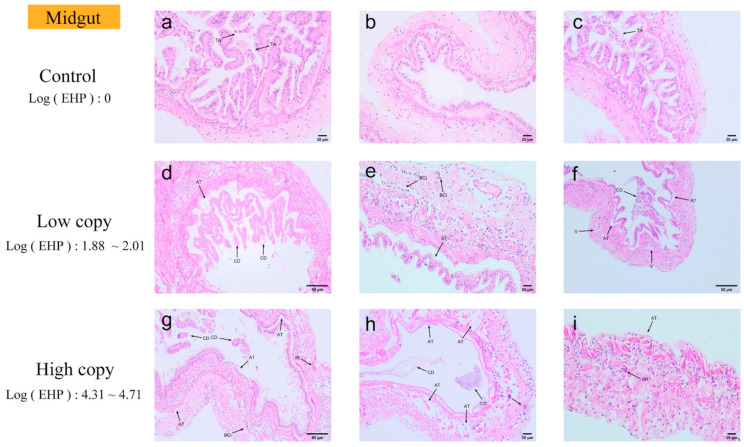
H&E staining of the midguts: (**a**–**c**) negative midgut; (**d**–**f**) low-load midgut; (**g**–**i**) high-load midgut. TA: epithelial cells detached from the basement membrane, and outer membrane detached from the muscle layer. CD: extensive necrosis of detached epithelial cells. BCI: blood cell infiltration. H: extensive cell necrosis forming vacuoles. IR: inflammatory response. BR: blackening reaction. S: microsporidian spores.

Midgut. The midgut appeared as a highly infected tissue in the quantitative experimental results (Figure 10). In midgut tissues with an EHP load of 0, minimal detachment of epithelial cells from the tissue was observed. In midgut tissues with low EHP loads, the detachment of epithelial cells from the basement membrane became more pronounced, accompanied by a significant number of necrotic epithelial cells. Vacuoles formed in numerous areas due to cell necrosis. Furthermore, hematopoietic infiltration began to emerge. The midgut tissue with high EHP loads exhibited more severe pathological changes, including the detachment of epithelial cells from the basement membrane and the separation of the outer membrane from the muscle layer. The lumen was filled with necrotic debris, and varying degrees of disintegration were observed in the epithelium, basement membrane, connective tissue, and muscle layers. As the infection progressed, the inflammatory response intensified, with hematopoietic and inflammatory cell infiltration observed in multiple areas, along with a melanotic reaction. Additionally, microsporidia were present in the highly infected midgut tissue.

**Figure 11:**
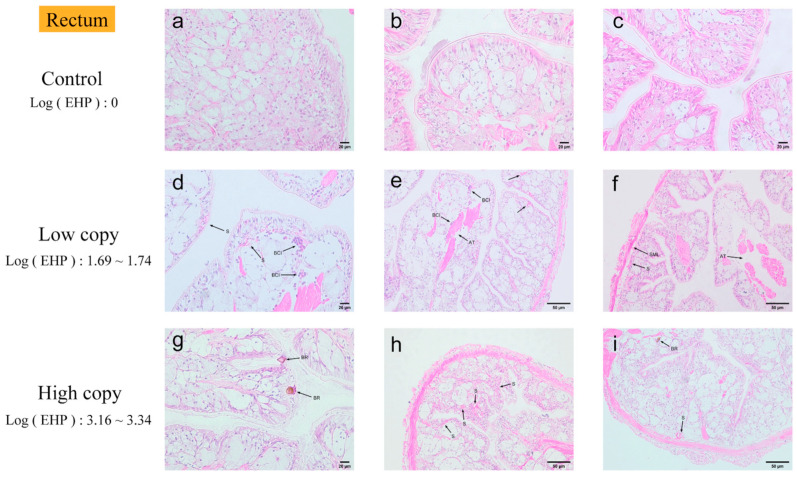
H&E staining of the rectums: (**a**–**c**) negative rectum; (**d**–**f**) low-load rectum; (**g**–**i**) high-load rectum. S: microsporidian spores. BCI: blood cell infiltration. AT: muscle tissue fiber rupture and detachment. SML: shedding of muscle layers. BR: blackening reaction.

Rectum. The quantitative results indicated that the rectum exhibited low susceptibility to EHP, with only mild tissue damage (Figure 11). In rectums with low EHP loads, microscopic observations revealed hematocyte infiltration in several regions of the tissue, along with the presence of microsporidia. Additionally, muscle fibers in the muscle layers were disrupted, and the detachment of the muscle layers was noted. In rectums with high EHP loads, a significant number of microsporidia were present in multiple regions, and the inflammatory response was more pronounced, accompanied by a melanotic reaction.

### 3.3. Histopathology Under Masson Staining

The results revealed the presence of microsporidia in nine different tissues, including the pyloric stomach, cardiac stomach, heart, gonads, midgut, hepatopancreas, rectum, gills, and tentacle gland tissues. Microsporidia were not detected in muscle tissue, likely due to the fact that the muscle fibers stained red with Masson’s stain, which closely matched the color of the spores, making them difficult to distinguish.

**Figure 12:**
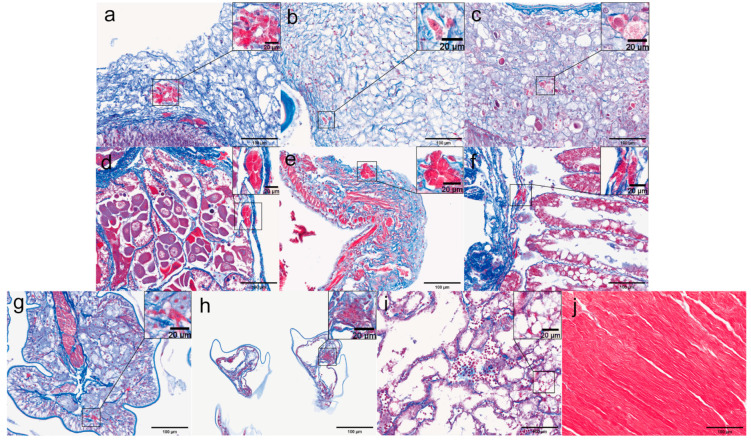
Microsporidia observed in various tissues under Masson staining: (**a**) cardiac stomach; (**b**) pyloric stomach; (**c**) heart; (**d**) gonads; (**e**) midgut; (**f**) hepatopancreas; (**g**) rectum; (**h**) gills; (**i**) antennal glands; (**j**) muscles.

Microsporidia stained red exhibited clear granularity and aggregation. Under Masson staining, aggregated microsporidia were primarily observed parasitizing the connective tissue, as well as some tissue boundaries. Notably, large aggregates of microsporidia were observed in the connective tissue of the cardia-gastric region (Figure 12a), with additional microsporidial clusters found at the interface between the epithelial cells and connective tissue. A similar pattern was noted in the pyloric stomach (Figure 12b). Various stages of microsporidia were observed within the connective tissue surrounding the heart (Figure 12c), where cavities had formed around the aggregated microsporidia. Additionally, microsporidian aggregates were present in the outermost connective tissue of the midgut (Figure 12e) as well as in the connective tissue of the rectum (Figure 12g). In the ovary (Figure 12d), microsporidial aggregates were identified in several locations within the ovarian wall. Microsporidia were difficult to observe in the hepatopancreas due to the purplish-red coloration of the renal tubules and were only detected in the peritoneal layer of the outer hepatopancreas. Microsporidia were also seen in the gill tissue (Figure 12h), specifically around the gill outlet vessels and within the cuticle. In contrast, the antennal gland tissue showed a less pronounced effect, with only a small number of red, granular microsporidia observed around the lining cells of the labyrinth epithelium. Muscle tissue (Figure 12j), being stained red, did not reveal any detectable microsporidia.

**Figure 13:**
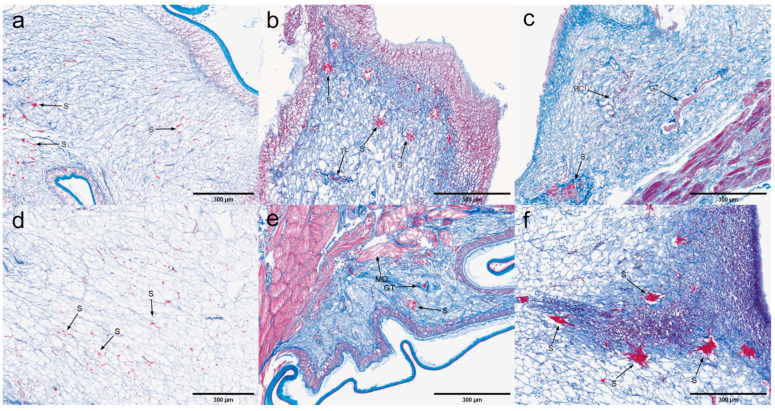
Pyloric stomach and cardiac stomach under Masson staining: (**a**–**c**) pyloric stomach; (**d**–**f**) cardiac stomach. S: microsporidian spores. TF: connective tissue fibrosis. BCI: blood cell infiltration. GT: granulation tissue in connective tissue. MD: muscle layer dissolution.

Pyloric and cardiac stomachs. Figure 13 shows the histological structure of the pyloric and cardiac stomachs under Masson staining. In the pyloric and cardiac stomachs with a copy number of 0 (Figure 13a,d), multiple scattered red areas were observed in the connective tissue, indicating the presence of microsporidia. Masson staining highlights fibrotic tissue in blue, with the fibrotic areas being stained dark blue by the aniline blue dye, allowing clear visualization of fibrosis in the connective tissue. For instance, in the low-copy pyloric stomach (Figure 13b), signs of tissue fibrosis are evident. Masson staining typically colors hemocytes, such as erythrocytes and leukocytes, red or light red due to the binding of acid magenta (Acid Fuchsin) dye. In the high-copy pyloric stomachs (Figure 13c), a substantial infiltration of hemocytes is observed within the connective tissue, with these cells appearing bright red or pink under staining. Granulation tissue typically appears red or purplish in Masson staining. In both high-copy pyloric and low-copy cardiac stomachs (Figure 13c,e), granulation tissue, indicative of acute inflammation or tissue repair following trauma, was observed. In regions of severe EHP infection, such as the low-copy pyloric and high-copy cardiac stomachs (Figure 13b,f), aggregates of microsporidia are evident. Masson staining can effectively stain muscle tissue red, with a lightening of the red color indicating myolysis. For instance, in the low-copy cardia stomachs (Figure 13e), areas of muscle tissue with lighter staining are indicative of myolysis. The above results indicate that a small amount of Microsporidium was still detected in the zero-copy pyloric and cardia stomach tissues, which did not produce detectable Cq values in the RT-qPCR assays.

**Figure 14:**
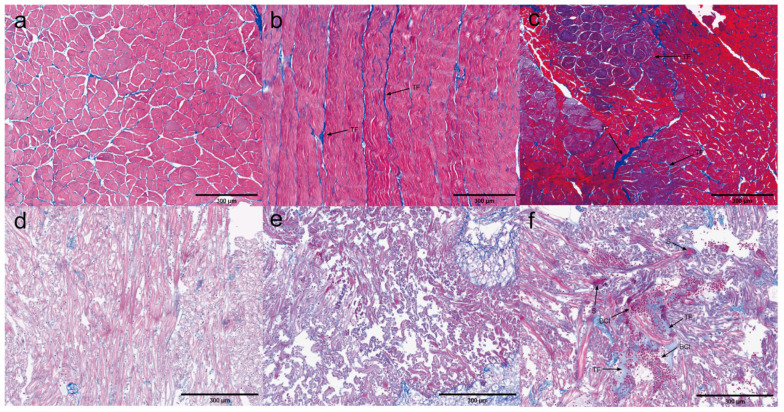
Muscle and myocardial tissue under Masson staining: (**a**–**c**) muscle; (**d**–**f**) myocardial tissue. TF: muscle tissue fibrosis. S: microsporidian spores. BCI: blood cell infiltration.

Muscle and myocardial tissue. Figure 14 illustrates the pathological features of muscle and myocardial tissue under Masson staining. Under normal conditions, muscle tissue appears red; however, in certain pathological states, collagen fiber deposition or hyperplasia may lead to a stronger blue coloration in the affected areas. Fibrosis of muscle tissue is observed in both low-copy and high-copy muscle samples (Figure 14b,c). As the degree of infection increases, the staining of myocardial tissue progressively changes, transitioning from the myocardium with a copy number of 0 (Figure 14d) to a deeper purple hue in both low-copy and high-copy myocardial tissues (Figure 14e,f). Furthermore, in high-copy myocardial tissue, significant infiltration of hemocytes and tissue fibrosis were observed, along with areas stained dark red, indicative of microsporidian aggregation. These pathological features were not as evident in H&E staining.

**Figure 15:**
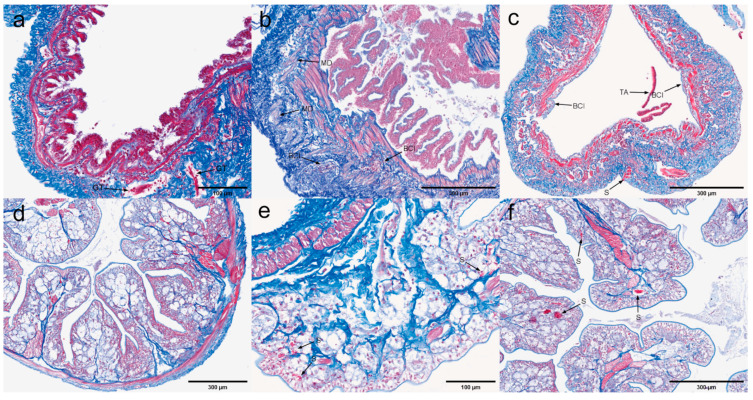
Midgut and rectum under Masson staining: (**a**–**c**) midgut; (**d**–**f**) rectum. GT: granulation tissue. MD: muscle layer dissolution. BCI: blood cell infiltration. TA: midgut epithelial detachment. S: microsporidian spores.

Midgut and rectum. Figure 15 displays the Masson staining of the midgut and rectum. Under Masson staining, granulation tissue was observed in the midgut with a copy number of 0 (Figure 15a). Meanwhile, in the low-copy midgut (Figure 15b), the staining of the longitudinal muscle appeared notably lighter, indicating myolysis. In the rectum (Figure 15e,f), which is considered a low-infection tissue, microsporidian aggregates were observed in the connective tissue under Masson staining. As the degree of infection increased, the distribution of microsporidia became more widespread, with larger clusters of aggregates.

**Figure 16:**
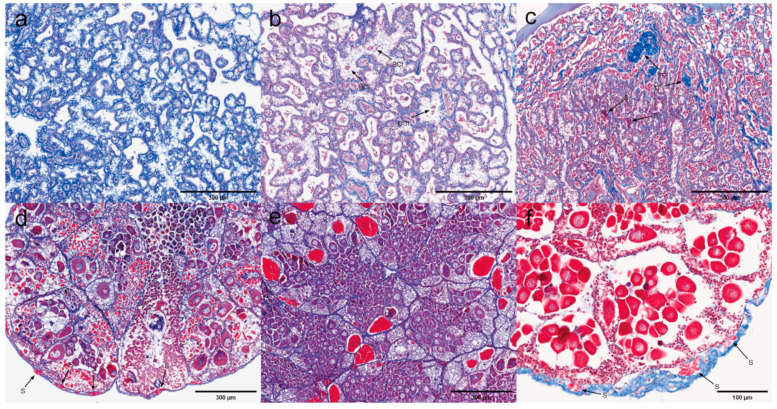
Antennal gland and gonad under Masson staining: (**a**–**c**) antennal gland; (**d**–**f**) gonad. BCI: blood cell infiltration. TF: tissue fibrosis. S: microsporidian spores.

Antennal gland and gonad. Figure 16 illustrates the pathological features of the antennal glands and gonads under Masson staining. In the antennal glands, the pathological changes became increasingly pronounced as the infection load rose. As the load progressed from 0 to low copy and high copy (Figure 16a–c), the staining shifted gradually from blue to purple. The degree of tissue fibrosis and hematopoietic cell infiltration deepened significantly, indicating the progressive nature of the lesions. Notably, in the high-copy antennal gland tissue (Figure 16c), the lesion area exhibited marked tissue fibrosis accompanied by microsporidian aggregation. In shrimp ovaries, staining variations were observed across different regions and developmental stages. These differences were attributed to factors such as cytoplasmic composition, lipid droplet content, nuclear morphology, and chromatin structure. Oocytes stained red presented challenges in distinguishing from microsporidian aggregation. However, microsporidial aggregation at the ovarian wall was clearly observed in ovarian tissues with zero and low-copy loads (Figure 16e,f).

**Figure 17:**
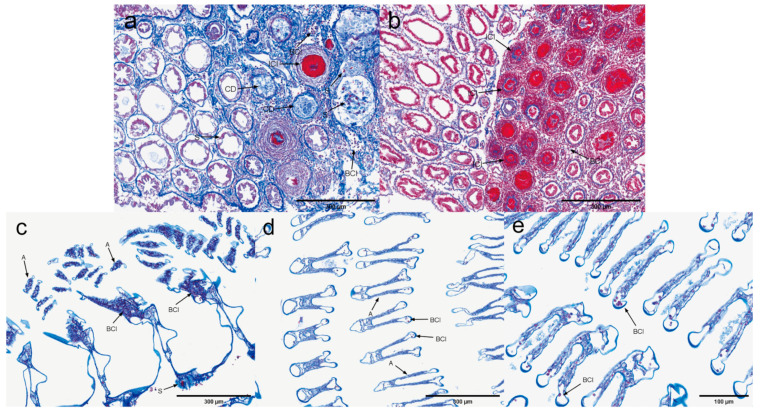
Hepatopancreas and gill under Masson staining: (**a**,**b**) hepatopancreas; (**c**–**e**) gill. ICI: inflammatory response with inflammatory cell infiltration. CD: cell necrosis with extensive cellular debris in the lumen. BCI: blood cell infiltration. S: microsporidian spores. TF: tissue fibrosis. A: gill filament atrophy.

Hepatopancrea and gill. Figure 17 illustrates the pathological features of the hepatopancreas and gills under Masson staining. The hepatopancreas exhibited more pronounced pathological changes, particularly in distinguishing between necrotic tissue fragments and microsporidian presence. Under Masson staining, necrotic tissue fragments were stained blue, while microsporidia appeared red. This distinction was clearly observed in low-copy hepatopancreatic tissue (Figure 17a). Multiple areas of intensified inflammatory response were observed in both low- and high-copy hepatopancreatic tissues (Figure 17a,b), where the mixture of inflammatory cells and collagen fibers caused a blending of red and blue staining, creating a stark contrast. Additionally, hematopoietic infiltration became more pronounced as the degree of infection increased. Under Masson staining, gill tissues primarily exhibited hemocyte infiltration and the atrophy of the gill filaments, with only a few microsporidian aggregates observed in gill tissues with zero loading (Figure 17c).

### 3.4. Histopathology Under Immunohistochemistry

Ten tissue samples were subjected to immunoassay using a rabbit-derived SWP1 protein polyclonal antibody as the primary antibody, and HRP-conjugated goat anti-rabbit IgG (H + L) as the secondary antibody, in order to accurately determine the localization of EHP expression within the tissues. Immunohistochemical analysis was conducted on both negative and positive tissue samples.

**Figure 18:**
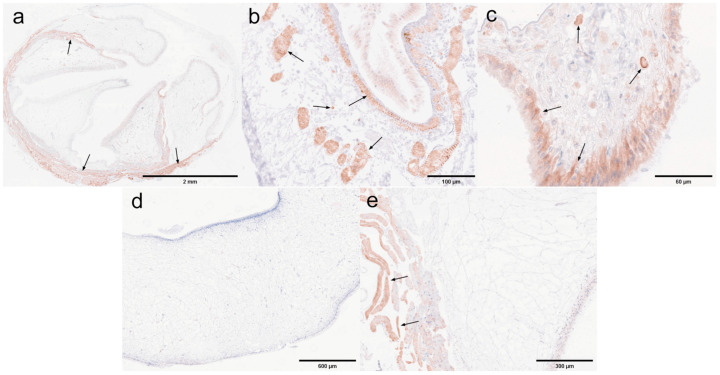
Pyloric and cardiac stomach under immunohistochemical staining: (**a**–**c**) pyloric stomach; (**d**,**e**) cardiac stomach. Arrows indicate positive signals.

Pyloric and cardiac stomachs. Dark brown precipitates indicated the presence of positive signals. The immunohistochemical results for the pyloric and cardiac stomachs are presented in Figure 18. In the pyloric stomach, both the negative sample (Figure 18a) and the positive sample (Figure 18b,c) displayed positive signals in the longitudinal and circular muscles. Additionally, positive signals were observed in the cytoplasm of epithelial cells and in the connective tissue of the positive pyloric stomach. In contrast, the negative cardiac stomach did not exhibit significant positive signals, while positive signals were detected in the muscularis propria of the positive cardiac stomach.

**Figure 19:**
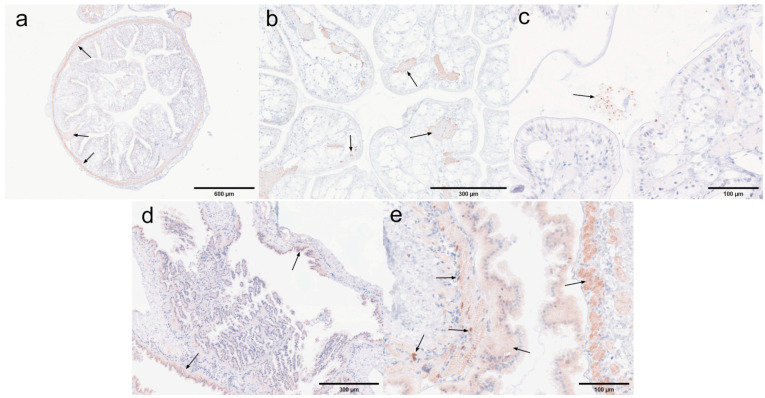
Rectum and midgut under immunohistochemical staining: (**a**–**c**) rectum; (**d**,**e**) midgut. Arrows indicate positive signals.

Midgut and rectum. The immunohistochemical results for the rectum and midgut are presented in Figure 19. In the low-infected rectum, the negative rectum (Figure 19a) already exhibited positive signals in the circular muscle layer. In the positive rectum (Figure 19b,c), positive signals were observed not only in the muscle tissue but also in the connective tissue, epithelial cells, and rectal lumen. In the susceptible midgut, weak positive signals were detected solely in the epithelial cells of the negative midgut (Figure 19d). However, in the positive midgut (Figure 19e), multiple distinct positive signals were noted in the circular muscle layer. Additionally, the positive signals in the epithelial cells of the midgut were restricted to the cytoplasmic region.

**Figure 20:**
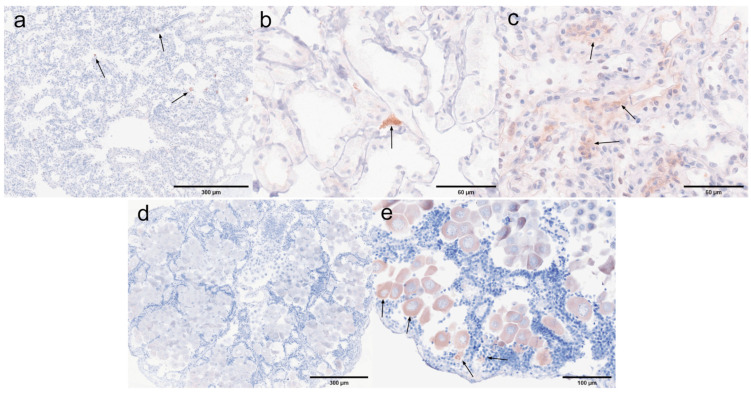
Antennal gland and gonad under immunohistochemical staining: (**a**–**c**) antennal gland; (**d**,**e**) gonad. Arrows indicate positive signals.

Antennal gland and gonad. The immunohistochemical results for the antennal glands and gonads are presented in Figure 20. Antennal glands play a critical role in the immune defense system of shrimp. Positive signals were observed in the coelomosac, labyrinth lumen, and labyrinthine cells in both negative (Figure 20a) and positive (Figure 20b,c) antennal glands. Immunohistochemical analysis of the ovaries revealed positive signals in the cytoplasm of the oocytes in the positive ovaries (Figure 20e). Additionally, positive signals were detected in the follicular cells surrounding the oocytes.

**Figure 21:**
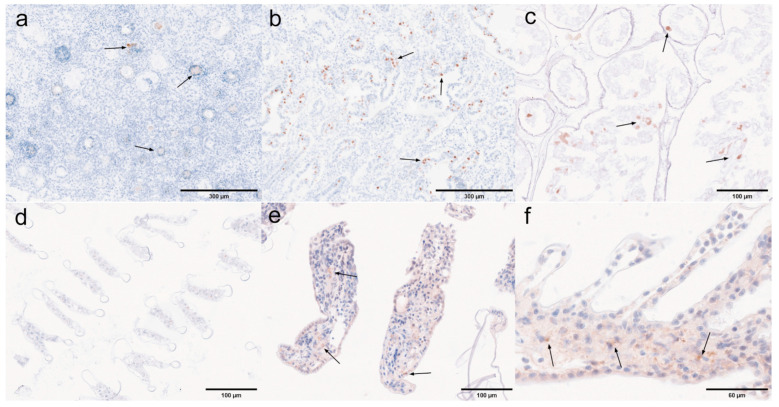
Hepatopancreas and gill under immunohistochemical staining: (**a**–**c**) hepatopancreas; (**d**–**f**) gill. Arrows indicate positive signals.

Hepatopancrea and gill. The immunohistochemical results for the hepatopancreas and gills are shown in Figure 21. While the distribution of microsporidia in hepatopancreatic tubules was difficult to observe using H&E and Masson staining, positive EHP signals were clearly detected through immunohistochemistry. In the positive hepatopancreas (Figure 21a–c), EHP positive signals were observed in multiple areas of shed and necrotic epithelial cells, as well as in the lumen of severely necrotic regions filled with cellular debris. Immunohistochemical analysis of the gills revealed no significant positive signals in the negative gills (Figure 21d), whereas in the positive gill tissues (Figure 21e,f), multiple granular dark brown deposits, indicative of positive signals, were observed.

**Figure 22:**
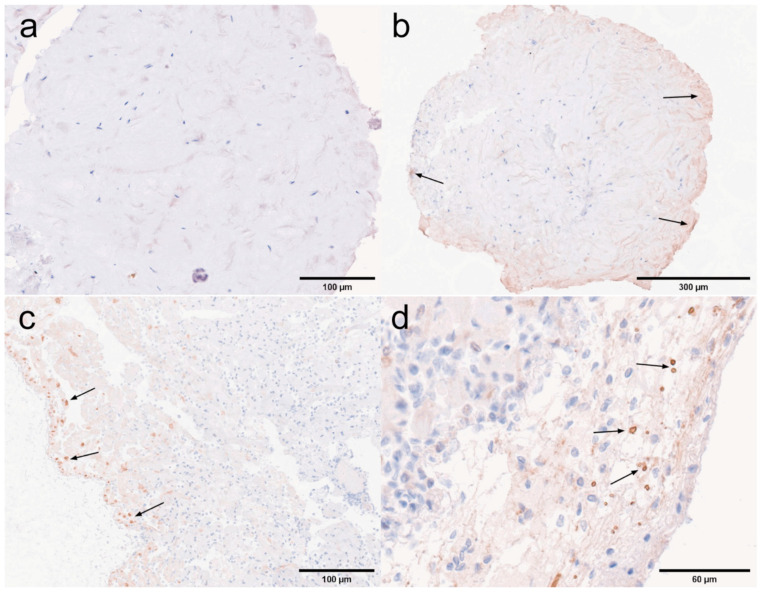
Muscle and myocardial tissue under immunohistochemical staining: (**a**,**b**) muscle; (**c**,**d**) myocardial tissue. Arrows indicate positive signals.

Muscle and myocardial tissue. The immunohistochemical results for muscle and cardiac muscle are shown in Figure 22. Based on the immunohistochemical analysis of other tissues, positive signals were clearly detected in the muscle layer of each tissue. In this study, muscle tissue from the first abdominal segment of the back of the *L. vannamei* was selected. No significant positive signals were observed in the negative muscle samples (Figure 22a), while only weak positive signals were detected in the positive muscle samples (Figure 22b). In myocardial tissue, prominent positive signals were observed both in the muscle tissue of the negative myocardium (Figure 22c) and in the connective tissue of the positive myocardium (Figure 22d). These findings suggest that EHP microsporidia tend to colonize the muscle tissue of the head.

### 3.5. Quality Control of Proteomics Data and Analysis of Sample Correlation

**Figure 23:**
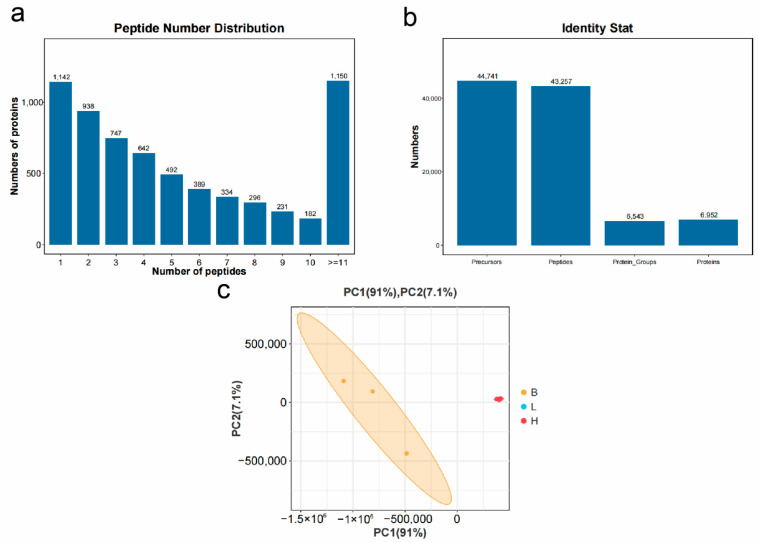
Protein identification results and sample correlation analysis: (**a**) peptide identification statistics; (**b**) protein identification statistics; (**c**) Principal Component Analysis of samples.

To ensure the reliability of the results, the protein identification analysis required that both peptide-level and protein-level data meet the identification criteria of Precursor Threshold 1.0% FDR and Protein Threshold 1.0% FDR. With a filtering criterion of FDR ≤ 0.01, a total of 44,741 spectra, 43,257 peptides, 6543 protein groups, and 6952 proteins were identified (Figure 23a). According to peptide count statistics, 82.55% of the identified proteins contained two or more unique peptides (Figure 23b). Principal Component Analysis (PCA) demonstrated that the control and experimental groups of *L. vannamei* samples were clearly separated into two distinct clusters in the PCA plot, indicating significant differences in expression patterns between the control and experimental groups. The low-copy and high-copy experimental groups showed minimal differences, resulting in overlap in the diagram.

### 3.6. Differentially Expressed Proteins

**Figure 24:**
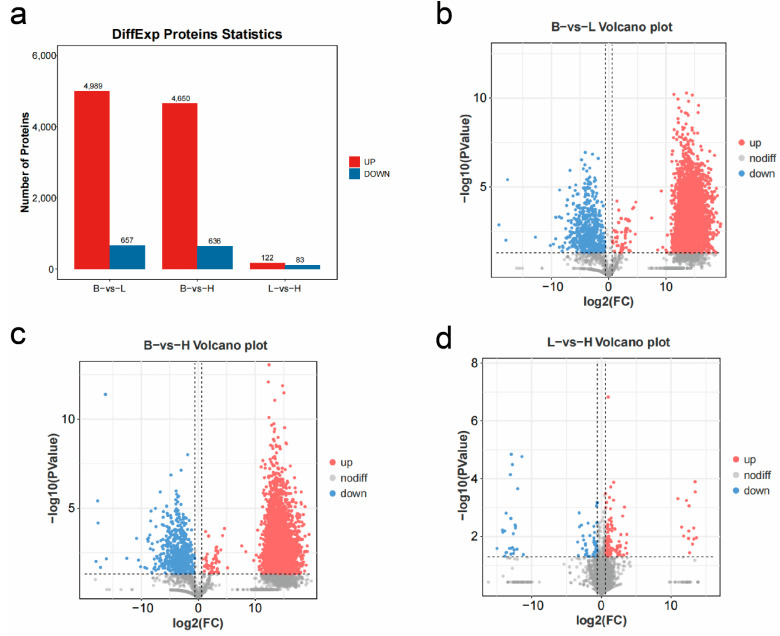
Results of protein differential analysis: (**a**) differential protein count between samples; (**b**–**d**) volcano plots of differential comparisons.

Differential analysis was conducted using |log_2_FC| > |log_2_1.5| and *p* < 0.05 as the threshold (Figure 24). In the comparison of B vs. L, the low-copy group identified 5646 DEPs, including 4989 upregulated and 657 downregulated proteins compared to the blank group. For the H vs. L comparison, the high-copy group revealed 5286 DEPs, with 4650 upregulated and 636 downregulated proteins relative to the blank group. In the B vs. H comparison, the high-copy group identified 205 DEPs compared to the low-copy group, of which 122 were upregulated and 83 were downregulated.

Some proteins with significant differential expressions were associated with immune responses and disease resistance. For instance, Cytochrome P450 2L1 (CYP2L1), TIMELESS interacting protein (TIPIN), serine palmitoyltransferase (SPTLC1), WASH complex subunit 1 (WASHC1), insulin receptor substrate 1 (IRS1), and ribosome biogenesis protein (BMS1) exhibited higher expression levels. Conversely, certain proteins showed lower expression levels, such as hemocyanin F chain (PPO2), eukaryotic translation initiation factor 2 subunit γ (EIF2S3), and E3 ubiquitin-protein ligase (RNF185).

### 3.7. Functional Enrichment Analysis of GO and KEGG Pathways

**Figure 25:**
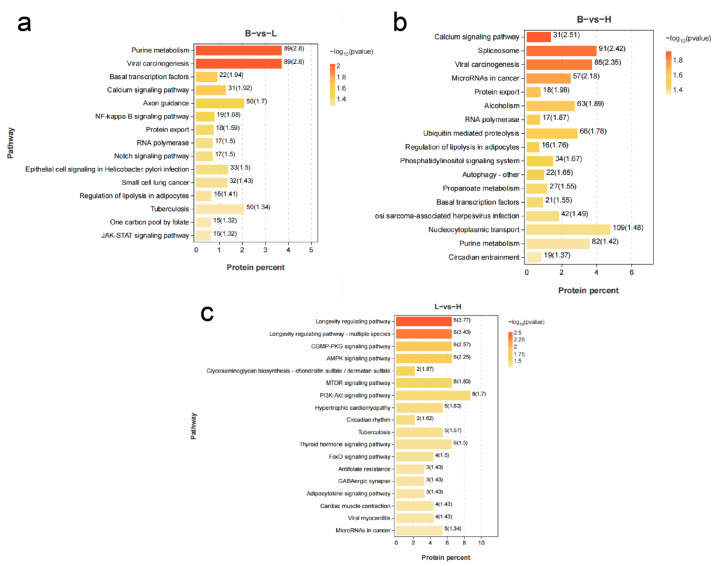
Bar chart of KEGG significant enrichment for each comparison group: (**a**) B vs. L; (**b**) B vs. H; (**c**) L vs. H.

The GO consists of three main ontologies describing the molecular function, cellular component, and biological process of genes. The results indicate that GO annotation analysis shows significant enrichment of DEPs across biological processes, molecular functions, and cellular components, with the most enrichment entries observed in biological processes and cellular components. As the focus of this study is on interpreting tissue pathology changes through bioinformatics, more attention was given to KEGG pathways and differentially enriched proteins in these pathways within the GO and KEGG pathway enrichment analysis. Using a *p*-value threshold of <0.05, 15 pathways were enriched in B vs. L, 17 pathways in B vs. H, and 18 pathways in L vs. H (Figure 25).

Enrichment analysis of KEGG pathways identified seven pathways potentially associated with hepatopancreatic histopathology: Phosphatidylinositol signaling system (ko04070), NF-kappa B signaling pathway (ko04064), Notch signaling pathway (ko04330), JAK-STAT signaling pathway (ko04630), propanoate metabolism (ko00640), regulation of lipolysis in adipocytes (ko04923), and autophagy—other (ko04136).

The Phosphatidylinositol signaling system is an essential intracellular secondary signaling system involved in numerous physiological and pathological processes within cells [13]. Among the DEPs, Phosphoinositide 3-kinase (PI3K) is a pivotal enzyme in this signaling pathway [14], with DEPs such as Phosphatidylinositol 3-Kinase Catalytic Subunit Type 3 (PIK3C3), Phosphatidylinositol 3-Kinase Catalytic Subunit Delta (PIK3CD), Phosphatidylinositol 3-Kinase Catalytic Subunit Type 2 Beta (PIK3C2B), and Phosphatidylinositol 3-Kinase Regulatory Subunit Alpha (PIK3R1) enriched within this pathway.

The NF-kappa B signaling pathway is an essential component of the immune system, involved in regulating immune and inflammatory responses, as well as the expression of genes related to cell proliferation and apoptosis [15]. In the results of this experiment, key DEPs enriched in this pathway include Prostaglandin-endoperoxide synthase 2 (PTGS2), kappa-B kinase alpha (IKKα), Myeloid Differentiation Primary Response Protein 88 (MyD88), and Interleukin-1 Receptor-Associated Kinase 4 (IRAK4).

The Notch signaling pathway primarily plays a role in regulating cell differentiation and maintaining tissue homeostasis [16]. Key DEPs enriched in this pathway include Neurogenic locus notch homolog protein 1 (Notch1), Presenilin 1 (PSEN1), Numb, TNF-α Converting Enzyme (TACE), and Histone Deacetylase 1 (HDAC1).

The JAK-STAT pathway is crucial for the signal transduction of many cytokines that regulate immune responses, such as interferons, interleukins, and growth factors [17]. DEPs enriched in this pathway include Son of Sevenless homolog 2 (SOS2), v-akt murine thymoma viral oncogene homolog 1 (Akt1), Suppressor of cytokine signaling 2 (SOCS2), Suppressor of cytokine signaling 7 (SOCS7), Mechanistic Target Of Rapamycin (mTOR), Signal transducing adapter molecule (Stam), and Signal transducer and activator of transcription 5B (Stat5b).

The propanoate metabolism pathway is associated with oxidative stress, particularly due to the production of reactive oxygen species (ROS) and reactive nitrogen species (RNS) within the metabolic process. When present in excess, these substances can damage cell structures and functions, leading to tissue pathological changes. Additionally, propanoate metabolism is closely linked to mitochondrial function, and metabolic abnormalities can result in mitochondrial dysfunction, affecting cellular energy metabolism and the apoptosis process. DEPs in this pathway include Acyl-CoA dehydrogenase, medium chain (ACADM), Acetyl-CoA synthetase short-chain family member 2 (ACSS2), and Acetyl-CoA synthetase short-chain family member 3 (ACSS3).

The regulation of lipolysis in adipocytes refers to the complex process by which adipocytes break down stored triglycerides into free fatty acids (FFAs) and glycerol, a process known as lipolysis [18]. Key DEPs enriched in this pathway include RAC-alpha serine/threonine-protein kinase (Akt1), G-protein alpha inhibitory subunit (Galphai), and Lipase, hormone-sensitive (LIPE), a gene that encodes hormone-sensitive lipase (HSL).

Finally, the autophagy pathway was also significantly enriched, playing a critical role in eliminating damaged organelles and protein aggregates [19]. The differentially enriched proteins in this pathway are primarily members of the ATG (Autophagy-related gene) protein family, including ATG3, ATG5, ATG6, ATG12, ATG13, ATG16, ATG4b, ATG2b, and ATG9b. The ATG protein family plays a crucial role in the cellular process of autophagy.

In the above pathways, the phosphatidylinositol signaling pathway and the autophagy pathway are enriched only in the B vs. H comparison, while the Notch signaling pathway, JAK-STAT pathway, and NF-κB signaling pathway are enriched only in the B vs. L comparison. The adipocyte lipolysis regulation pathway and propanoate metabolism pathway are enriched in both the B vs. H and B vs. L groups.

## 4. Discussion

### 4.1. Assessment of EHP Infection Extent Across Different Tissues

The results of the present study indicated variation in EHP infection across different tissues and organs of *L. vannamei*. The hepatopancreas exhibited the highest detection rate and infection load, followed by the pyloric stomach. The cardiac stomach, situated near the pyloric stomach, exhibits a slightly lower level of infection compared to the pyloric stomach. Furthermore, significant infection levels were observed in the anterior, middle, and posterior segments of the midgut. Beyond the digestive tract tissues mentioned, the muscle, gills, and tentacle glands also exhibited high detection rates and infection loads. Detection rates in other tissues remained below 50%, indicating that the tissues most susceptible to EHP infection in *L. vannamei* are the hepatopancreas, pyloric stomach, cardiac stomach, midgut, muscle, gills, and antennal glands.

Karthikeyan et al. observed mild banding in the eyestalks when detecting EHP by PCR [20]. Our research findings indicate that a detection rate of 34.45% was observed in the eyestalk, which implies that, although the detection rate in the eyestalk is less than half of the overall rate, it remains an optimal organ for the non-lethal detection of EHP via RT-qPCR. Evidence indicates that EHP can be transmitted vertically from infected broodstock to their offspring [21]. In the current study, EHP was also detected in the gonads of *L. vannamei*, with a detection rate of 27.27% and an average logarithmic load of 2.19. These findings suggest that EHP infects the gonads of shrimp and may facilitate vertical transmission to progeny. The antennal glands serves as a crucial excretory organ in shrimp, functioning analogously to the kidney in vertebrates [22]. Liu et al. identified the shrimp antennal glands as a key infection site for WSSV invasion [23]. Consequently, it is suspected that EHP may also have the potential to infect the antennal glands of shrimp. The results of this experiment indicated that the antennal glands exhibited a high detection rate of 50.91%, signifying their susceptibility to EHP infection.

The same tissue was analyzed for EHP loading across three different states of shrimps, including surviving, dying, and dead. The results demonstrate a clear positive correlation between the health status of shrimp and the EHP load. As the health of the shrimp deteriorates, both the detection rates and the EHP loads in various tissues increase, with the highest values observed in dead shrimp. This trend highlights the significant impact of health status on the distribution and intensity of EHP infection across different tissues in shrimp. There was an overall increase in EHP detection rates and loads in dead shrimp compared to dying and surviving shrimp, indicating accelerated microsporidian proliferation in deceased shrimp.

EHP primarily parasitizes the hepatopancreatic ducts of prawns [3]. The results of this study indicated a detection rate of 100% for the hepatopancreas, which also exhibited the highest loads. Infections in the pyloric stomach, cardiac stomach, and the anterior and posterior segments of the midgut and rectum, which are additional components of the digestive system, were also analyzed. The pyloric and cardiac regions of the stomach were observed to be interconnected; however, a significant disparity in EHP load was noted between them, with the pyloric stomach exhibiting a considerably higher load than the cardiac stomach. This difference may be attributed to their distinct functions: the pyloric stomach is primarily involved in the physical and chemical breakdown of food, while the cardiac stomach focuses on filtering chyme and compressing solid matter.

### 4.2. Tissue Analysis by H&E, Masson Staining, and Immunohistochemistry

Chaijarasphong et al. identified pathological changes in diseased hepatopancreatic tissues, which were primarily characterized by significant thinning of renal tubular epithelial cells and a substantial number of exfoliated cells within the tubular lumen [24]. Tourtip et al. conducted the first study on the histology and cytopathology of EHP-infected *L. vannamei*, observing that EHP spores were distributed within an eosinophilic structure in the cytoplasm of R, B, and E cells in the hepatopancreatic tubular epithelium [7]. In this experiment, the hepatopancreas of the shrimp, identified as the primary parasitic tissue, exhibited a 100% infection rate and demonstrated the most significant tissue damage. The primary observations included hepatopancreatic atrophy, loss of hepatic tubule structure, inflammatory response, and melanization, with no microsporidia detected. This may be due to the insufficient staining of the microsporidia. The immune defense of the gills is crucial during the initial stages of pathogenic bacterial infections. Histopathological studies conducted by Janakiram et al. on cultured samples of shrimp, *L. vannamei*, concurrently infected with abdominal segment deformity disease (ASDD) and EHP, revealed significant vacuolization in the gills [25]. In this experiment, vacuolization was observed in the gills of EHP-infected shrimp. Additionally, gill filament atrophy, hemocyte infiltration, and microsporidia were noted. Microsporidia at different developmental stages exhibited a bright pink coloration in the gill filaments. Intriago et al. observed spore accumulation in the anterior cephalothorax muscles near the antennal gland tubules and in the dorsal cephalothorax muscles of *L. vannamei* [26]. Additionally, Wang et al. studied microsporidiosis in *Exopalaemon carinicauda* and reported a substantial presence of microsporidia in the muscle, along with a disorganized arrangement of muscle filaments and fragmented muscle fibers [10]. In this experiment, muscle tissues exhibited primarily fiber rupture and striated muscle dissolution under H&E staining. The absence of microsporidia may be attributed to possible missed detection under the microscope and the degree of staining. The intestine and hepatopancreas are critical organs for nutrient absorption and immune function in shrimp [27], and they are also primary sites of EHP infection. Tang et al. similarly identified EHP in the midgut epithelial cells of *L. vannamei* samples infected with EHP, using histological techniques and ISH [28]. In the H&E-stained histopathological images of the midgut, we observed significant tissue damage, primarily manifested as the detachment of intestinal epithelial cells. However, no presence of microsporidia was detected. There are few histopathological studies on the EHP infection of tissues such as the heart, gonads, antennal glands, stomach, and rectum. Both low-infection and susceptible tissues exhibited varying degrees of tissue damage. In some tissues, such as the rectum and stomach, the presence of microsporidia was observed, particularly in the pyloric and cardiac stomachs. Microsporidia were observed within the connective tissue of both the pyloric and cardiac regions of the stomach. Tangprasittipap et al. utilized H&E staining on heavily infected *L. vannamei* shrimp and found that EHP was not readily detectable through this method. However, EHP was clearly identified using in situ hybridization [29]. Due to the small size of the microsporidia spores, they appeared pink under H&E staining, slightly darker than the surrounding cytoplasm, but were not easily distinguishable. The results of H&E staining in this study also indicated that microsporidial aggregates were difficult to detect in the hepatopancreas with more advanced stages of infection, as the microsporidia were often missed using this technique. In conclusion, the histopathological analysis of various tissues under H&E staining primarily exhibited inflammatory responses and cellular degeneration. Microsporidia were observed in only a few tissues, which may be attributed to three factors, including the faint staining of microsporidia under H&E staining, insufficient magnification under the microscope, and interference from background coloration.

In contrast, Chang et al. demonstrated that Masson staining provided a clear delineation of the overall shape of shrimp hepatopancreas sporocysts. The large number of spores within the sporocysts exhibited strong granularity in the host cells, with the spores staining in shades of toffee or magenta, making the sporocysts more easily observable [30]. Therefore, Masson staining was employed in this study to investigate the distribution of microsporidia across different tissues. Firstly, we conducted preliminary experiments to examine the staining effect of Masson staining in various tissues. Given the high detection rate of EHP microsporidia with Masson staining, this experiment further enhanced the pathological analysis of EHP-infested tissues across various conditions. The pathological characteristics of the tissues under no loading, low loading, and high loading conditions were compared using Masson staining.

According to the histopathological analysis of Masson staining, this method demonstrates a clear advantage over H&E staining in detecting EHP infection across multiple tissues. Firstly, the detection rate of microsporidia is higher with Masson staining compared to H&E staining, and microsporidia are more likely to proliferate in the connective tissue and structural walls of various groups. Additionally, Masson staining reveals pathological changes more clearly in muscle and myocardial tissues, especially in the phenomenon of muscle fibrosis, where the extent of fibrosis increases with the severity of EHP infection. Notably, unlike muscle tissue, myocardial tissue exhibits more specific staining characteristics. This is due to the small amounts of connective tissue and collagen fibers present in the myocardium alongside muscle fibers. In certain pathological conditions, the proliferation of fibrous tissue leads to an interweaving of myocardium and connective tissue (such as collagen fibers), resulting in a purple staining reaction.

Furthermore, in several tissues, such as the myocardium, antennal glands, and hepatopancreas, the infiltration of blood cells is more pronounced under Masson staining. The images show a large number of blood cells, with staining shifting from blue to purplish-red. However, areas with severe lesions were often characterized by focal infiltration of numerous inflammatory cells, particularly leukocyte such as neutrophils, which typically stained red. This made it challenging to distinguish microsporidia within the inflamed regions. Masson staining may have potential applications in the study of ovarian development in shrimp, as oocytes at different stages exhibit distinct staining patterns, aiding in the identification of ovarian developmental progression. However, despite the use of optical or specialized microscopes to examine tissue samples infected with microsporidia, there remains a risk of missed detection. The detection of EHP spores using light microscopy is challenging due to the requirement for high magnification lenses. A more sensitive and specific detection approach involves targeting specific genes or proteins associated with EHP [31]. Therefore, immunohistochemistry was employed to target and localize EHP within tissues.

Unlike H&E and Masson staining, immunohistochemistry allows for precise localization of EHP expression in affected areas, offering high specificity and sensitivity. In this experiment, positive signals were detected in all ten tissues, and microsporidia at different developmental stages were observed. Mature spores appeared as brown, round, or oval structures under the microscope, while secondary spores and meronts formed multiple small brown particles within the cell. The plasmodium was uniformly distributed in the cytoplasm, staining it brown. The immunohistochemical results revealed that EHP proliferates in the cytoplasm of various tissues, such as in the epithelial cells of the stomach and intestine and the cytoplasm of connective tissue, with prominent positive signals. And EHP is prone to proliferation in connective and muscle tissues, particularly in head muscle tissues, with a high susceptibility to infection, such as in the muscular layers surrounding the stomach and intestine. Masson and H&E staining could not confirm whether Microsporidia infected the oocytes; however, immunohistochemical analysis of the ovaries revealed positive signals in the cytoplasm of the oocytes in the positive ovaries, providing evidence for the vertical transmission of EHP.

In this study, we employed three methods, H&E staining, Masson staining, and immunohistochemistry, to investigate the histopathology of EHP infection in *L. vannamei*. H&E staining is a simple, rapid, and cost-effective technique commonly used in routine pathological examination. It effectively reveals fundamental pathological change. However, H&E staining lacks molecular or antigenic specificity and does not allow for the clear identification of microsporidia. In contrast, Masson staining effectively labels collagen fibers, making it ideal for observing pathological fibrosis. Additionally, it has the advantage of staining microsporidia in a distinct red color, facilitating the assessment of their distribution. However, Masson staining is more complex and time-consuming than H&E staining, and like H&E staining, it lacks molecular specificity, providing no detailed molecular information about microsporidia. Although immunohistochemistry allows for precise localization of EHP target sites, it is complex to perform and associated with high costs. Notably, in the quantitative results of the three methods, microsporidia were observed in EHP-negative tissue samples, indicating that the sensitivity of RT-qPCR for EHP detection is insufficient. Therefore, in the pathogen detection of shrimp hepatopancreatic microsporidia, molecular quantitative methods should be combined with histopathological examination to improve diagnostic accuracy. Additionally, in the results of H&E and Masson staining, we observed tissue damage in some negative tissue samples. This may be due to prior EHP infection, which was resolved through the immune regulatory response of the shrimp, but tissue damage persists.

### 4.3. Differential Proteins and KEGG Pathways Related to Histopathology

In the DEP analysis, significant upregulation of cAMP response element-binding protein (CREB) family members CREB1, CREBBP, CREBL2, and Protein Kinase A Catalytic Subunit Alpha 1 (PKA-C1) was observed. PKA (protein kinase A) and CREB are two key signaling molecules that play a crucial role in regulating melanin synthesis. Additionally, several key proteins in the PI3K family were also significantly upregulated, promoting the expression of enzymes associated with melanin synthesis and thereby regulating melanin production. The upregulation of these DEPs may be related to the observed histopathological finding of localized darkening in certain areas of the liver-pancreas region.

Research on the Phosphatidylinositol signaling system pathway has highlighted its critical role in cellular immunity in crustaceans. In crustaceans, studies demonstrated that PI3K expression is a key protein in cellular immunity. Kong et al. found that LvPI3K was strongly upregulated in *L. vannamei* following infection with *Vibrio alginolyticus* [32]. Furthermore, a report by Hou et al. in 2020 indicated that EsPI3K significantly increased in Eriocheir sinensis after *V. alginolyticus* infection [33]. In histopathology, the role of PI3K is primarily reflected in the activation of hemocytes and phagocytes, as well as autophagy. The activation of hemocytes and phagocytes manifests in hepatopancreatic tissue pathology as hemocyte and inflammatory cell infiltration, with focal aggregation of large numbers of inflammatory cells in severely affected areas. Excessive autophagy can also lead to tissue atrophy, which is manifested in the hepatopancreas as hepatopancreatic atrophy in histopathology. Related DEPs include PIK3C3, which initiates and maintains autophagy by generating phosphatidylinositol 3-phosphate (PtdIns3P) [34]. Additionally, Pik3cd (Phosphoinositide-3-kinase catalytic subunit delta) is involved, which can regulate the expression of various intracellular signaling molecules, such as caspase-3, that play a critical role during the inflammatory process of cells. In the DEPs analysis, we found that caspase-3 was also significantly upregulated. Caspase-3 is an important cysteine-aspartic protease that plays a critical role in apoptosis. The study by Liu et al. found that the expression level of the caspase-3 gene is associated with EHP infection. In this experiment, caspase-3 was detected as a DEP [35], providing evidence for the relationship between caspase-3 gene expression and EHP infection.

In invertebrates, the innate immune system is divided into humoral immunity and cellular immunity, with the NF-kappa B signaling pathway being indispensable for humoral immunity. LvNKRF has been reported by researchers to be induced by WSSV infection. In recent years, the NF-kappa B signaling pathway has been recognized as a major regulatory mechanism of immune response in shrimp [36]. One of the critical functions of MyD88 is its ability to activate NF-κB, which is a central factor in regulating inflammatory responses, as well as in the regulation of antimicrobial peptide (AMP) production, activation of immune cells, and modulation of the inflammatory response to infections. In the screening of DEPs, we indeed found that anti-lipopolysaccharide factors (ALFs) were significantly upregulated. This protein is an antimicrobial peptide that specifically inhibits bacterial outer membrane components, such as lipopolysaccharides, and helps prevent bacterial invasion. However, it remains unclear whether it can defend against EHP. The other three differentially expressed key proteins, PTGS2, IKKα, and IRAK4, are all related to inflammation production [37]. Excessive production and release of inflammatory factors can lead to tissue cell damage and death, resulting in the disruption of tissue structure and function. In the histopathology of the hepatopancreas infected with EHP, this is manifested by the extensive infiltration and focal aggregation of inflammatory cells, accompanied by significant necrosis and shedding of renal tubular epithelial cells.

The Notch signaling pathway involves numerous proteins that regulate cell fate. For example, Neurogenic locus Notch homolog protein 1 (Notch1), a key protein in this pathway, plays a critical role in cell fate determination. Histopathological analysis revealed significant upregulation of apoptosis-related proteins, indicating that this pathway contributes to tissue pathology by promoting cell death, as evidenced by the extensive shedding and death of cells within the hepatopancreatic tubules. Moreover, this pathway may play a role in shrimp immunity and disease resistance against EHP infection by activating specific immune cells. However, the overactivation of the Notch signaling pathway may lead to excessive proliferation of immune cells, resulting in immune dysregulation, which can subsequently cause chronic inflammation or tissue damage. The Notch signaling pathway is also involved in tissue repair processes during tissue injury or pathogenic infection in shrimp. TACE plays a key role in the inflammatory response and is secreted by immune cells. It activates tumor necrosis factor-alpha (TNF-α), which, in turn, induces the production of other pro-inflammatory cytokines and chemokines [16]. These factors further recruit more immune cells to the site of inflammation, amplifying the inflammatory response and resulting in the significant infiltration of inflammatory cells in the hepatopancreatic tissue, leading to tissue pathology. Additionally, TNFAIP8, a protein associated with TNF-α, was identified in the DEPs of both the low and high copy groups. It was first discovered in response to TNF-α stimulation.

Some STAT proteins within the JAK-STAT pathway can induce apoptosis, which plays a crucial role in eliminating damaged cells. In infected liver/pancreatic tissue pathology, a large number of necrotic cells have been observed, leading to a significant upregulation of STAT proteins. The suppressor of cytokine signaling (SOCS) family includes two DEPs in this pathway: SOCS2 and SOCS7. Members of the SOCS family play a critical role in regulating the function of immune cells, including macrophages and other immune cells [17,38]. mTOR is a highly conserved serine/threonine protein kinase within cells. Its enrichment in this pathway suggests that EHP invasion of liver/pancreatic tissues causes tissue damage, with increased activity in processes such as autophagy and cellular metabolism.

In the propionate metabolism pathway, the proteins ACADM, ACSS2, and ACSS3 are essential for ATP production. These three proteins are crucial for ATP production. In energy-demanding tissues like the liver–pancreas, disturbances in energy metabolism can lead to a series of pathological changes, such as triggering apoptosis or necrosis [39]. Furthermore, mitochondrial dysfunction can lead to increased production of reactive oxygen species (ROS), which, in turn, induces oxidative stress and inflammatory responses.

The enrichment of lipolysis regulation pathways in adipocytes is also a factor influencing the liver/pancreatic tissue pathology. Akt1 is a crucial regulator of cell survival, primarily promoting cell survival by inhibiting the activation of pro-apoptotic molecules such as Caspase-9. In our differential protein screening, we observed significant upregulation of Caspase-9, Caspase-3, and Caspase-6. This suggests that the cells are damaged, with the precursor Caspase-9 being activated, subsequently triggering downstream executioner caspases, such as Caspase-3 and Caspase-6, which ultimately lead to cell death [40]. Pathologically, this results in the appearance of numerous necrotic cells in the liver-pancreas tissue. Galphai is primarily involved in the chemotaxis of immune cells, driving their migration to areas of inflammation or injured tissue to combat infection. In EHP infection of liver–pancreas tissue, this induces tissue inflammation, leading to a significant upregulation of Galphai. HSL is an important enzyme involved in the hydrolysis and metabolism of lipids. The overactivation of lipolysis can lead to tissue damage and fibrosis. The increase in FFAs can activate immune cells, such as macrophages, leading to an intensified inflammatory response that may further impair tissue function [41]. Fibrosis in the infected liver/pancreatic tissues can be observed through Masson staining. Additionally, liver/pancreatic cells are capable of storing and metabolizing lipids. After EHP infection of the liver–pancreas, damage to these tissues may affect lipid metabolism, thus activating the lipolysis regulatory pathways in adipocytes.

**Figure 26:**
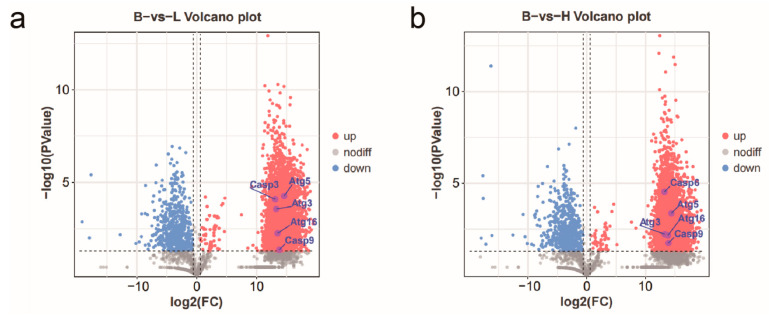
Position of Caspase protein family and ATG protein family in volcanic map: (**a**) B vs. L; (**b**) B vs. H.

In the autophagy pathway, ATG proteins play a crucial role in immune responses. They assist in the clearance of intracellular pathogens such as bacteria and viruses [42], suggesting that the significant upregulation of ATG proteins may play a pivotal role in combating EHP infection. Additionally, EHP infection leads to extensive cellular necrosis, which necessitates autophagy to degrade and recycle damaged or unnecessary intracellular components, including proteins and organelles [19]. We identified two proteins that may play a role in the defense against EHP infection in *L. vannamei*, as shown in Figure 26. Firstly, certain members of the Caspase protein family, involved in both the adipocyte lipolysis regulation pathway and the Phosphatidylinositol signaling system, exhibited regulatory activity in the hepatopancreas after EHP infection. Microsporidia grow by invading host cells and utilizing the host’s metabolic resources, during which the host cell’s Caspase proteins may be activated, triggering apoptosis. Microsporidia typically complete their lifecycle by proliferating within host cells. If the host cell undergoes early death or significant apoptosis, the proliferation of the microsporidia may be impaired. Additionally, in the autophagy pathway, the significant upregulation of the ATG protein family may also be associated with resistance to EHP infection. EHP microsporidia may be engulfed by host cells through the autophagy pathway and transported to the lysosome for degradation, thereby resisting EHP infection by directly phagocytosing the pathogen. Proteins such as ATG3 and ATG5 may also regulate the cell’s life and death fate by interacting with the Caspase family, which is associated with apoptosis [43].

In histopathology, pathological changes are often accompanied by significant alterations in the expression of specific proteins. Proteomic analysis can identify these proteins that are either upregulated or downregulated in affected tissues, thereby helping to explain the morphological and functional changes observed in cells during pathological examination. Analysis of the seven main signaling pathways revealed that most abnormalities involve disruptions in inflammation, immune regulation, metabolism, and autophagy pathways. Furthermore, from the perspective of proteomics analysis of pathological changes, we identified two protein families, Caspase and ATG, which may be key proteins involved in the resistance to EHP.

## 5. Conclusions

This study identifies key sites such as the hepatopancreas, stomach, and midgut, as well as lesser-studied tissues like the eyestalk and gonads. Histopathological examination using H&E, Masson staining, and immunohistochemistry revealed the presence of microsporidia in non-hepatopancreatic tissues. Proteomic analysis revealed disruptions in inflammation, immune regulation, metabolism, and autophagy, shedding light on the molecular mechanisms of EHP-induced tissue damage and informing future disease management strategies. It is known that EHP is widely present in non-hepatopancreatic tissues. In the future, it is expected that the infection of EHP in hemolymph and other tissues will be detected, enabling non-invasive and rapid diagnosis of EHP through hemolymph detection. Additionally, the proteins potentially associated with anti-EHP activity need to be validated.

## Figures and Tables

**Table 1:** EHP loadings in each tissue across the three different states.

Tissue	Living Shrimp		Moribund Shrimp		Dead Shrimp	
	Average Load	Detection Rate	Average Load	Detection Rate	Average Load	Detection Rate
Hepatopancreas	3.71	100%	3.69	100%	4.52	100%
Eyestalk	1.65	28%	1.82	35%	2.50	46%
Gill	1.05	56%	2.45	53%	2.45	85%
Antennal gland	2.00	44%	1.95	47%	2.60	69%
Heart	1.89	36%	1.57	17%	2.03	46%
Muscle	2.31	72%	2.41	65%	2.71	85%
Gonad	2.28	28%	1.41	18%	2.53	38%
Pyloric stomach	3.23	84%	3.51	82%	4.11	92%
Cardia stomach	2.62	80%	2.65	76%	3.25	92%
Anterior midgut	3.58	84%	2.84	65%	3.32	85%
Middle midgut	3.11	76%	2.81	59%	3.62	69%
Posterior midgut	3.00	60%	2.42	59%	3.42	69%
Rectum	2.06	48%	1.47	12%	2.63	69%

## Data Availability

The data presented in this study are available on request from the corresponding author. The protein data is currently being uploaded to the database.
